# Mechanistic roles and therapeutic potential of bacteriophages in inflammatory gastrointestinal diseases

**DOI:** 10.20517/mrr.2025.62

**Published:** 2025-11-12

**Authors:** Baiyun Ding, Mingze Fan, Yong-Peng Shi, Xingyao Chen, Yi Duan

**Affiliations:** ^1^State Key Laboratory of Immune Response and Immunotherapy, Department of Infectious Diseases, The First Affiliated Hospital of USTC, Center for Advanced Interdisciplinary Science and Biomedicine of IHM, Division of Life Sciences and Medicine, University of Science and Technology of China, Hefei 230026, Anhui, China.; ^2^Key Laboratory of Anhui Province for Emerging and Reemerging Infectious Diseases, Hefei 230027, Anhui, China.

**Keywords:** Gut virome, bacteriophages, phage therapy, digestive disease

## Abstract

The gut virome, particularly its viral and phage components, is increasingly recognized as a key modulator of intestinal microbial dynamics in gastrointestinal inflammatory diseases. Beyond well-characterized bacterial dysbiosis, growing evidence suggests that virome alterations contribute to the development and progression of inflammatory bowel disease, metabolic dysfunction-associated steatohepatitis, alcoholic hepatitis, primary sclerosing cholangitis, primary biliary cholangitis, and pancreatitis. As the most abundant viruses in the gut, bacteriophages influence microbial ecosystem stability and host immune responses through lytic and lysogenic interactions with bacterial populations. Amid the growing burden of multidrug-resistant infections and heightened interest in microbiota-based interventions, phage therapy has re-emerged as a viable strategy in both preclinical and translational contexts. This review synthesizes recent insights into bacteriophage dynamics in the context of major gastrointestinal and hepatopancreatic inflammatory diseases, highlighting potential compositional shifts, proposed mechanisms of phage–microbe interactions, and supportive evidence from animal models and early clinical applications. We also discussed the critical challenges that had to be addressed to enable clinical translation, including host range restrictions, resistance and safety concerns, immunogenicity, and delivery limitations, while emphasizing emerging strategies such as phage engineering, encapsulation technologies, and standardized regulatory frameworks.

## INTRODUCTION

The human gastrointestinal tract harbors a complex and dynamic microbial ecosystem, commonly referred to as the gut microbiota, which plays a fundamental role in maintaining host metabolic, immune, and epithelial homeostasis^[1-5]^. Recent studies have demonstrated that disruptions in the gut microbiota are closely associated with a wide range of gastrointestinal and hepatobiliary inflammatory diseases. These include inflammatory bowel disease (IBD), liver disorders such as metabolic dysfunction-associated steatohepatitis (MASH) and alcoholic hepatitis (AH), biliary tract diseases such as primary sclerosing cholangitis (PSC) and primary biliary cholangitis (PBC), as well as pancreatitis^[6-15]^. While microbial dysbiosis in these conditions has traditionally been attributed to bacterial imbalances, accumulating evidence suggests that the gut virome - particularly its bacteriophage constituents - may also play a pivotal role in disease onset and progression^[16-20]^. 

The intestinal virome refers to the complete assemblage of viruses inhabiting the gastrointestinal tract. This includes eukaryotic viruses (e.g., *Adenoviridae*, *Herpesviridae*), archaeal viruses, and bacteriophages (phages), which specifically infect bacterial hosts^[21-23]^. Among these, phages constitute the most abundant and genetically diverse viral group. Through lytic and lysogenic interactions, they shape bacterial community composition, mediate horizontal gene transfer, and influence host immune responses^[24-26]^. Recent advances in metagenomics and viromics have revealed that phages are not merely passive residents but active ecological players in gut microbial homeostasis and immune modulation^[27-30]^. These findings support the emerging view of bacteriophages as critical regulators within the gut ecosystem.

Although bacteriophages were first discovered over a century ago and initially explored for their antibacterial potential, their therapeutic application was largely abandoned following the advent of broad-spectrum antibiotics and limited understanding of microbial ecology^[31-35]^. However, the global rise in multidrug-resistant (MDR) infections and renewed appreciation of host-microbiota-virus interactions have reignited scientific and clinical interest in phage-based therapies^[36,37]^. Supported by breakthroughs in molecular biology, structural virology, and high-throughput sequencing technologies, bacteriophages are now recognized as promising tools for targeted microbiota modulation and precision antimicrobial strategies^[38-40]^. This review synthesizes recent findings on bacteriophage dynamics in the context of IBD, MASH, AH, PSC, PBC, and pancreatitis. We examine emerging therapeutic applications of phages, highlighting proposed mechanisms, translational opportunities, and current limitations in clinical development. Literature was searched in PubMed, Scopus, and Web of Science for studies published between 2015 and 2025 using the terms “bacteriophage”, “phage therapy”, “gut virome”, and “inflammatory 

gastrointestinal diseases”.

## BACTERIOPHAGES IN INFLAMMATORY GASTROINTESTINAL DISEASES

**Figure 1:**
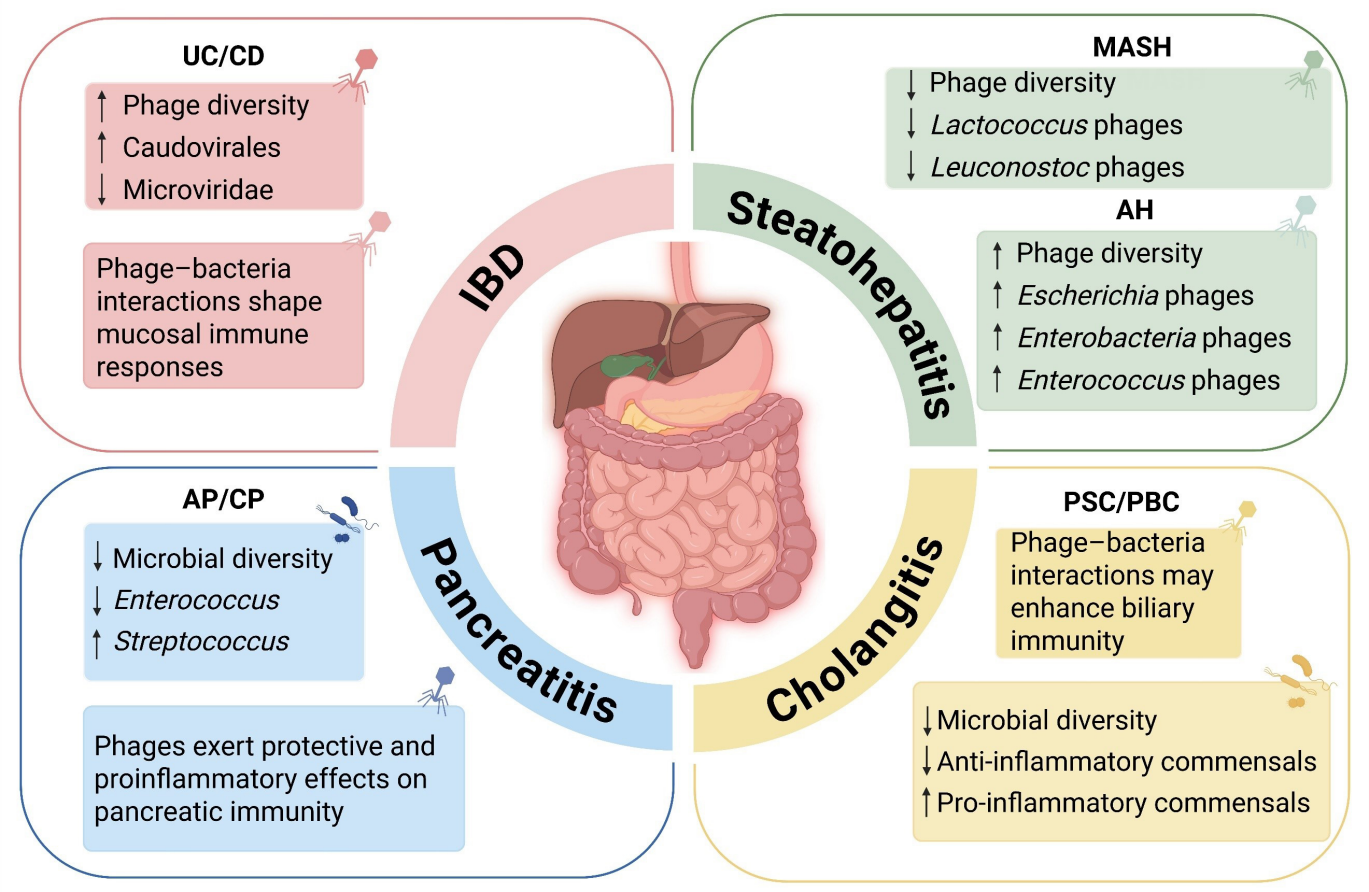
Disease-specific alterations and immunomodulatory roles of gut bacteriophages in inflammatory gastrointestinal diseases. In IBD, an increased abundance of *Caudovirales* and decreased *Microviridae* are observed, alongside enhanced mucosal immune engagement. In MASH, reduced *Lactococcus* and *Leuconostoc* phages correlate with liver fibrosis severity. In AH, phages targeting pathobionts such as *Escherichia* and *Enterococcus* are enriched. Although direct virome evidence remains limited in PSC and PBC, prominent microbial dysbiosis and immune perturbations suggest potential contributions of phage-bacteria interactions to biliary inflammation. In both acute and chronic pancreatitis, bacteriophages modulate immune responses bidirectionally by influencing bacterial lysis and TLR9 signaling. Created with BioRender.com. Data summarized from published studies^[49-52,61-65,90,91]^. UC: Ulcerative colitis; CD: Crohn’s disease; IBD: inflammatory bowel disease; MASH: metabolic dysfunction-associated steatohepatitis; AH: alcoholic hepatitis; PSC: primary sclerosing cholangitis; PBC: primary biliary cholangitis; AP/CP: acute/chronic pancreatitis; TLR9: toll-like receptor 9.

In healthy individuals, a dynamic equilibrium between bacteriophages and their bacterial hosts maintains ecological homeostasis and contributes to both immune and metabolic regulation^[41]^. However, accumulating evidence indicates that this balance is frequently disrupted in a range of inflammatory conditions affecting the gut, liver, and pancreas. These disease states are often characterized by reduced phage diversity, shifts in phage-bacteria composition, and altered interactions at the phage–microbe–host interface^[42,43]^. This section reviews the ecological functions of gut-associated bacteriophages under physiological conditions, summarizes reported patterns of phage-related alterations in inflammatory diseases, and sets the stage for disease-specific discussions that follow [Figure 1].

### Inflammatory bowel disease

IBD, which includes ulcerative colitis (UC) and Crohn’s disease (CD), is characterized by chronic, relapsing inflammation of the gastrointestinal tract. UC is primarily limited to continuous mucosal inflammation in the colon, while CD features patchy inflammation and increased intestinal permeability throughout the gut^[44-46]^. The multifactorial pathogenesis of IBD involves genetic susceptibility, mucosal immune dysregulation, epithelial barrier dysfunction, and gut microbiota imbalances^[47,48]^.

In addition to these well-characterized pathogenic mechanisms, virome-wide association studies (VWAS) have identified disease-specific alterations in the gut viral community, particularly among bacteriophages. Patients with IBD often display increased overall viral diversity and elevated levels of phages, especially those belonging to the order *Caudovirales*, alongside reductions in *Microviridae*^[8,49,50]^. In CD, *Caudovirales* phages have been shown to positively correlate with Enterobacteriaceae and Pasteurellaceae, and negatively with Bacteroidaceae, mirroring patterns of bacterial dysbiosis^[51,52]^. These compositional shifts suggest selective enrichment of phages associated with inflammatory pathobionts.

Mechanistic studies further support the involvement of bacteriophages in mucosal inflammation. Transfer of virus-like particles (VLPs) from IBD patients into germ-free mice increased susceptibility to chemically induced colitis, suggesting a direct immunomodulatory effect of gut-resident viruses^[53]^. Moreover, bacteriophage infection of *Bacteroides fragilis* has been shown to influence phase-variable DNA inversion systems, modulating capsular polysaccharide expression and thereby altering host-microbe immune interactions^[54]^. 

Clinical observations offer preliminary support for virome-targeted interventions. In patients with UC, fecal microbiota transplantation (FMT) not only restored viral diversity but also introduced donor-derived temperate phages, such as those integrated within vitamin B-producing Oscillospiraceae, suggesting a potential role in reestablishing gut immune and metabolic balance^[50]^. Beyond these clinical observations, recent studies have begun to explore whether combining FMT with bacteriophage-based strategies may enhance therapeutic efficacy. One approach involved the administration of targeted phages prior to FMT to eliminate pathogenic bacteria and facilitate donor microbiota engraftment. Another strategy focused on supplementing FMT with lytic phages after transplantation to suppress residual or recurring pathobionts and improve the durability of treatment. Although these combined approaches remained in the early stages of investigation, they offered a promising direction for optimizing IBD therapy by integrating microbial replacement with precision-targeted viral modulation^[50,55]^. 

In contrast, analysis of ileal mucosal samples from CD patients revealed decreased levels of both lytic and temperate phages, particularly those targeting *Bifidobacterium* and *Bacteroides*. Transferring CD-derived VLPs into murine models exacerbated colitis severity, underscoring the pathogenic potential of virome perturbation^[30]^. These findings raised the possibility that host innate immune sensing of altered viral components mediates disease progression. Recent studies have identified endosomal toll-like receptor 9 (TLR9) as a key sensor of 5′-C-phosphate-G (CpG)-rich phage or viral DNA that triggered a myeloid differentiation factor-88 (MyD88)-nuclear factor-kappa B (NF-κB) → interleukin-6 (IL-6)/signal transducer and activator of transcription 3 (STAT3) inflammatory cascade. In preclinical models, reducing luminal viral DNA attenuated TLR9 signaling and mitigated colitis, whereas exogenous CpG restored the inflammatory response and the TLR9 antagonist oligodeoxynucleotide 2088 (ODN-2088) reversed it^[56,57]^. 

### Steatohepatitis

MASH and AH are two major subtypes of inflammatory liver disease, both characterized by hepatocellular injury, immune activation, and microbial dysbiosis^[58,59]^. MASH, formerly known as non-alcoholic steatohepatitis (NASH), is marked by hepatic steatosis, lobular inflammation, and fibrosis, often occurring in the context of obesity, insulin resistance, and gut barrier dysfunction^[60]^. A virome study revealed that patients with histologically advanced MASH exhibited reduced phage diversity (*P* = 0.005), particularly among *Lactococcus* and *Leuconostoc* phage (*P* ≤ 0.05). These losses correlated with greater hepatic inflammation and fibrosis, and incorporating viral metrics improved clinical risk stratification^[17]^. Complementary data showed that even low-level alcohol intake in MASH patients induces a shift in the phageome toward an AUD-like profile, with selective re-expansion of *Lactococcus* phages, suggesting convergence between metabolic and alcohol-related liver injury^[61]^. In a high-risk population study, increased prevalence of Crassvirales phages targeting *Prevotella*, and temperate phages infecting *Escherichia*, was linked to hepatic steatosis, further supporting a role for phage-bacteria interactions in shaping liver outcomes^[62]^. These findings collectively underscore the potential involvement of gut bacteriophages in the inflammatory processes underlying MASH.

AH is an acute and often life-threatening form of alcohol-related liver disease, characterized by jaundice, hepatocyte necrosis, and systemic inflammation^[63]^. Recent metagenomic research has begun to elucidate the role of the gut virome in disease progression. A key multicenter study analyzing fecal viromes from 89 AH patients, alongside cohorts of individuals with alcohol use disorder (AUD) and healthy controls, revealed a striking increase in phage diversity within the AH group, particularly among bacteriophages targeting *Escherichia*, *Enterobacteria*, *Enterococcus*, and *Staphylococcus*. Concurrently, enrichment of phages targeting beneficial taxa such as *Lactococcus* led to marked depletion of these bacteria, reflecting a broader phage-bacteria imbalance^[61,64]^. Expansion of phages targeting lactate-producing bacteria depleted these commensals, weakening colonization resistance and IgA-mediated niche control. The resulting Enterococcus/Enterobacteriaceae blooms and endotoxin translocation amplified TLR-dependent inflammatory signaling and activated hepatic innate immunity, consistent with the severe immune phenotype of AH^[64,65]^. Collectively, these findings define an “AH virome signature” associated with more severe disease phenotypes.

Although emerging evidence has revealed virome alterations in MASH and AH, the functional implications of phage community shifts for gut-liver axis inflammation remain undefined. Critical mechanistic gaps persist in experimental models, particularly regarding whether specific phage-bacteria interactions exert direct effects on hepatic immune activation. In future studies, elucidating these interactions will be essential for establishing the immunopathogenic relevance of the virome in liver inflammation.

### Cholangitis

PSC and PBC are chronic cholestatic liver diseases that, despite differing in etiology, share common features such as immune dysregulation, microbial imbalance, and disruption of the gut-liver axis^[66-68]^. 

PSC is characterized by inflammation and fibrosis of both intrahepatic and extrahepatic bile ducts, with a notable co-occurrence of UC in nearly 70% of patients^[69]^. Studies consistently report a reduction in microbial diversity and selective expansion of taxa such as *Veillonella*, *Enterococcus*, and *Klebsiella*, which are thought to compromise intestinal barrier integrity and trigger hepatic immune responses^[70]^. Although the virome associated with PSC remains incompletely defined, recent metagenomic surveys have revealed a pronounced enrichment of *Klebsiella pneumoniae* (*K. pneumoniae*) and *Enterococcus gallinarum* (*E. gallinarum*), both of which correlate with disease severity and adverse outcomes. In susceptible murine models, colonization with PSC-derived *K. pneumoniae* strains promotes bacterial translocation, mesenteric lymph node colonization, and robust hepatic Th17 activation - hallmarks of exacerbated cholangitis^[71]^. Altogether, these findings highlight the potential for disrupted bacteriophage-bacterium networks to drive microbial overgrowth and immune dysregulation in PSC.

In contrast, PBC is an autoimmune-mediated cholangiopathy marked by progressive loss of small intrahepatic bile ducts and is closely associated with antimitochondrial antibody (AMA) production^[72,73]^. Although ursodeoxycholic acid (UDCA) confers clinical benefit, a substantial proportion of patients fail to achieve complete biochemical remission and may ultimately progress to cirrhosis or require liver transplantation^[74,75]^. Microbiome analyses in untreated PBC patients have revealed reduced bacterial diversity and a shift toward pro-inflammatory taxa such as *Enterococcus*, *Streptococcus*, and *Klebsiella*, alongside depletion of anti-inflammatory commensals such as *Faecalibacterium* and *Ruminococcaceae*^[76]^. Although direct virome profiling in PBC remains lacking, similarities in microbial disruption across PSC and other inflammatory liver diseases suggest that changes in bacteriophage populations may likewise contribute to disease progression. Supporting this hypothesis, preclinical models have demonstrated that gut-derived bacterial translocation, which is facilitated by bile acid-induced epithelial injury, can promote Th17-mediated hepatic inflammation^[71,77,78]^. These studies suggest that phage-bacteria interactions may influence immune responses in cholangitis. Future research should focus on using disease-specific models to conduct more in-depth investigations and uncover mechanisms that are distinct from those seen in other types of liver inflammation.

### Pancreatitis

Pancreatitis, encompassing both acute (AP) and chronic (CP) forms, is a significant inflammatory disorder of the pancreas that frequently co-occurs with gut dysbiosis and barrier dysfunction^[79-82]^. Classically, gut dysbiosis in AP and CP is associated with reduced microbial diversity and the expansion of opportunistic species such as *Enterococcus* and *Streptococcus*, which can compromise intestinal integrity and promote microbial migration to pancreatic tissue^[83-85]^. While bacterial disruptions in these conditions have been extensively characterized, the role of the intestinal virome - particularly bacteriophages - has only recently gained attention. Emerging evidence suggests that shifts in phage populations may modulate microbial community dynamics, facilitate bacterial translocation, and influence host immune responses during pancreatitis^[86-89]^. The virome may represent an additional regulatory layer over these microbial ecosystems and their inflammatory sequelae.

Experimental studies in AP mouse models have shown that depletion of the enteric virome alters disease severity. Mice treated with an oral antiviral cocktail (ribavirin, lamivudine, and acyclovir) showed reduced pancreatic inflammation and tissue injury. Mechanistically, this protection coincided with attenuated TLR9 signaling, which is known to respond to viral DNA fragments, including those derived from bacteriophages^[90]^. Furthermore, phage-mediated bacterial lysis may release immunogenic bacterial components that shape local and systemic immune responses. For example, prophage induction during early inflammation could lead to the formation of immunostimulatory aggregates that amplify innate immune activation^[91]^. These findings suggest that bacteriophages may exert multifaceted effects on gut barrier integrity, microbial translocation, and inflammatory progression in pancreatitis.

Virome alterations in acute and chronic pancreatitis may both reflect and reshape local microbial ecosystems and immune activity. Unlike better-studied gut-liver interactions, the gut-pancreas axis remains poorly characterized in this context. Future research should emphasize longitudinal sampling in well-phenotyped patient cohorts, alongside experimental models that capture the interplay between phage-induced microbial turnover and pancreatic inflammation. A more detailed understanding of these processes may ultimately support phage-guided diagnostics or microbiota-based interventions tailored to pancreatitis.

## PHAGE-BASED THERAPY IN GASTROINTESTINAL INFLAMMATION

Phage therapy, the therapeutic use of bacteriophages to eliminate pathogenic bacteria, has existed for over a century. However, with the discovery and widespread adoption of antibiotics, its clinical application declined significantly, particularly in Western countries. In recent years, mounting concerns over antibiotic resistance have fueled renewed interest in phage-based strategies. Lytic phages remain effective against MDR strains, as bacterial resistance to antibiotics does not equate to resistance against phage-mediated lysis. Their unique ability to selectively target bacterial species or strains, self-replicate at infection sites, and exert minimal off-target effects makes them an attractive alternative or a complement to traditional antibiotics^[92]^.

In the field of gastroenterology, phage therapy was initially explored for classic infectious diseases such as cholera and bacterial diarrhea^[93]^. Its scope has since expanded to include a range of chronic and inflammatory disorders. Current research has investigated phage applications in diverse contexts [Table 1], including targeting adherent-invasive *Escherichia coli* (AIEC) in CD^[94]^, and *in vitro* killing of *Clostridioides difficile* (*C. difficile*) using phage tail-like particles (tailocins, PTLPs)^[95]^, eliminating high-alcohol-producing *K. pneumoniae* in MASH^[28]^, and modulating *Enterococcus faecalis* (*E. faecalis*) in alcohol-associated hepatitis^[29]^. Taken together, these studies underscore the favorable safety profile and translational potential of phage therapy in managing gastrointestinal and hepatobiliary disease.

**Table 1:** Summary of phage-based therapeutic interventions in inflammatory gastrointestinal diseases

**Disease**	**Study Year(s) ** **and Location**	**Status**	**Population and Treatment Method**	**Dose and Frequency**	**Outcome**	**Reference**
CD	2019; New York and Baltimore, Maryland, USA	Clinical trial, active	30 patients with CD in remission Oral administration	EcoActive (phage cocktail) twice daily for 15 days	N/A	[94]
CD	2020; Milan, Italy	Clinical case (compassionate use)	57-year-old woman with CD, with multisite infection (gastrointestinal tract, urinary tract,* etc.*) Oral and intrarectal	One phage (10^7^ PFU orally, administered every 12 h for 3 weeks; 10^6^ PFU intrarectally, once daily for 2 weeks)	The original host (*K. pneumoniae*) was no longer detected	[96]
UC	2015; Hershey, Pennsylvania, USA	Preclinical	Clinical *Clostridioides difficile* isolates (primarily ribotype 027) *In vitro* treatment	N/A (*in vitro*)	PTLPs exhibited bactericidal activity against 21 of 25 ribotype 027 strains without affecting non-clostridial species, demonstrating strain-specific efficacy and high target selectivity *in vitro*	[95]
Acute bacterial diarrhea	2009-2011; Dhaka, Bangladesh	Clinical trial	120 boys with diarrhea Oral administration	11 T4-like phages (3.6 × 10^8^ PFU) or ColiProteus (1.4 × 10^9^ PFU), 3 times daily for 4 days	Safe but lack of efficacy	[97]
MASH	2023; Beijing, China	Preclinical	Male mice were colonized with high-alcohol-producing (HiAlc) *K. pneumoniae* Oral administration	Phage phiW14 (10^4^-10^6^ PFU) or imipenem (200 µg/200 µL), once daily for 1, 4, or 7 days	Lytic phages targeting HiAlc K. pneumoniae have been shown to effectively and safely reverse diet-induced MASH and restore metabolic homeostasis.	[28]
AH	2019; San Diego, California, USA	Preclinical	Humanized mice were colonized with cytolysin-producing *E. faecalis* strains isolated from patients with AH Oral administration	Phage cocktail (3 or 4 phages) 10^10^ PFU, administered once 1 day before sacrifice	Humanized mice colonized with cytolysin-producing *E. faecalis* showed reduced hepatic inflammation and injury after treatment with phage cocktails	[29]
PSC	2023; Tokyo, Japan	Preclinical	Specific-pathogen-free mice prone to hepatobiliary injury were colonized with PSC-patient-derived *K. pneumoniae* Oral and Intravenous	A 4-phage cocktail (10^9^ PFU orally in 200 μL buffer, administered every 3 days; 10^8^ PFU intravenously in 100 μL buffer, administered every 3 days)	PSC mice showed reduced liver inflammation and disease severity after treatment with phage cocktails	[70]
Pancreatitis	2017; San Diego, California, USA	Clinical case (compassionate use)	68-year-old man with necrotizing pancreatitis complicated by pancreatic pseudocyst Intracavitary and intravenous	Nine phages in 3 cocktails (5 ×10^9^ PFU intravenously, administered every 6-8 h; 5 ×10^9^ PFU intracavitary, administered every 6-12 h)	Patient completely recovered	[98]

CD: Crohn’s disease; UC: ulcerative colitis; MASH: metabolic dysfunction-associated steatohepatitis; AH: alcoholic hepatitis; PSC: primary sclerosing cholangitis; PBC: primary biliary cholangitis; AP/CP: acute/chronic pancreatitis; *K. pneumoniae*: *Klebsiella pneumoniae*; *E. faecalis*: *Enterococcus faecalis*; *A. baumannii*: *Acinetobacter baumannii*; *C. difficile*: *Clostridioides difficile*; AIEC: adherent-invasive *Escherichia coli*; PTLP: phage tail-like particle; PFU: plaque-forming unit.

### IBD

Bacteriophage therapy has emerged as a promising and precision-targeted strategy for modulating gut microbial communities in IBD, which includes UC and CD. Traditional treatments for IBD, such as immunosuppressants and biological agents, primarily focus on dampening inflammatory responses. However, they do not directly address the underlying dysbiosis that characterizes the gut microbiota in these disorders^[99]^. Phage therapy, by contrast, enables selective depletion of pathogenic bacteria without affecting the broader microbial ecosystem, thereby offering a disease-modifying approach rather than a purely symptom-suppressive one. In the context of UC, growing evidence supports the relevance of the gut virome, particularly bacteriophages, in shaping disease course and therapeutic outcomes. FMT has shown clinical efficacy in a subset of UC patients, and virome analysis suggests that donor-derived bacteriophages may be key effectors in this process. For example, Majzoub *et al.* utilized metagenomic sequencing in two randomized controlled FMT trials and identified a specific temperate phage integrated within an Oscillospiraceae bacterium that persisted in the recipient gut post-FMT and was associated with clinical remission^[50]^. This finding not only underscores the role of bacteriophages in disease modulation but also highlights the potential of specific phage-host symbioses in restoring mucosal homeostasis. The integration of bacteriophages into beneficial bacterial taxa may act as a stabilizing force that promotes the recovery of microbial diversity and barrier function. These integration events can modify local transcription through promoter insertion, transcriptional read-through, or disruption of small RNAs. At the same time, they may introduce “moron” genes encoding toxins, adhesins, restriction-modification systems, or metabolic enzymes, collectively reshaping host bacterial virulence and metabolism^[100]^. In certain contexts, these changes may confer beneficial effects; for example, prophages integrated into Oscillospiraceae have been linked to clinical remission in UC. Here, lysogeny may attenuate pathogenic traits, promote the production of vitamins and anti-inflammatory metabolites, or disrupt bacterial virulence programs, thereby supporting mucosal homeostasis. Because outcomes are strain- and locus-dependent, resolving prophage architecture with high-resolution tools such as long-read sequencing or proximity ligation methods will be essential to anticipate and harness these potentially advantageous effects^[101]^.

In CD, one of the most well-characterized microbial contributors to pathogenesis is AIEC, a strain capable of invading epithelial cells, surviving within macrophages, and triggering chronic inflammation through the release of pro-inflammatory cytokines such as tumor necrosis factor alpha (TNF-α) and IL-6. A growing number of preclinical studies have investigated phage therapy targeting AIEC, demonstrating significant reductions in colonization and intestinal pathology in mouse models of colitis. Among these, the EcoActive**^TM^** phage cocktail, composed of 7 lytic phages specifically engineered to cover a wide spectrum of AIEC strains, has shown over 95% *in vitro* efficacy and has now entered a Phase 1/2a clinical trial to evaluate its safety, tolerability, and potential efficacy in CD patients^[94]^. These translational efforts underscore the feasibility of phage therapy as a clinically scalable intervention. Beyond *Escherichia coli *(*E. coli*), other pathobionts such as *K. pneumoniae* have been increasingly implicated in IBD, particularly due to their capacity for mucosal colonization, antibiotic resistance, and pro-inflammatory potential^[49]^. A five-phage combination therapy has been shown to effectively reduce *K. pneumoniae* burdens in murine colitis models while concurrently alleviating histopathological signs of inflammation^[55]^. Importantly, this phage mixture retained activity against both antibiotic-sensitive and -resistant strains, highlighting its utility in the context of rising antimicrobial resistance. This line of research also emphasizes that phage therapy need not be restricted to a single pathogen but can be designed to target multiple contributors to dysbiosis and inflammation.

*Clostridioides difficile* infection (CDI) is a clinically significant complication in IBD, particularly triggered by antibiotic exposure or immunosuppressive therapy^[102]^. Because strictly lytic phages against *C. difficile* remain scarce and many described isolates are temperate, recent work has shifted toward phage-derived agents. These include endolysins (phage-encoded peptidoglycan hydrolases) and PTLPs, which can kill *C. difficile* without requiring phage replication^[95,103]^. These phage-derived biomolecules offer a novel route to selectively eliminate *C. difficile* populations while avoiding the broader ecological disruptions often caused by antibiotics. Meanwhile, given that antibiotics remain the standard of care, adjunctive regimens combining vancomycin or fidaxomicin with targeted phage-derived agents, such as endolysins or PTLPs, should be evaluated. Such phage-antibiotic synergy may enhance pathogen clearance and lower relapse rates in recurrent CDI^[103]^. Furthermore, they present an attractive therapeutic option for recurrent or refractory CDI, a clinical scenario with limited treatment alternatives.

### Steatohepatitis

Phage-targeted modulation of gut-derived bacterial contributors has been extensively evaluated in preclinical models of MASH. Among the most consistently implicated microbial drivers is endogenous ethanol-producing *K. pneumoniae*, which generates significant quantities of ethanol through fermentation pathways, thereby promoting hepatocellular injury. Experimental transplantation of fecal microbiota from human donors harboring high-alcohol-producing *K. pneumoniae* strains into germ-free mice has been shown to faithfully replicate multiple pathological features of steatohepatitis. These include macrovesicular lipid accumulation in hepatocytes, lobular infiltration by inflammatory cells, and elevated levels of serum aminotransferases, reflecting hepatic damage. Remarkably, pretreatment of the donor fecal samples with lytic phages specifically designed to target and eliminate the ethanol-producing *K. pneumoniae* strains resulted in complete prevention of disease onset in recipient mice. This phage-mediated decolonization strongly supports a direct causal role for microbial ethanol production in MASH pathogenesis. It also highlights the capacity of bacteriophages to interrupt this pathological axis at its microbial source. Supporting this observation, therapeutic efficacy has been validated in animal models, where oral administration of a *K. pneumoniae*-specific lytic phage led to reduced steatosis, suppressed hepatic pro-inflammatory responses, and normalization of liver function biomarkers. Notably, these therapeutic benefits were achieved without significantly altering the broader gut microbial community structure, nor were there signs of systemic or local toxicity, underscoring the high degree of target specificity and safety inherent to phage therapy^[28]^. In parallel to these targeted strategies, Fecal virome transplantation (FVT), which involves transferring purified bacteriophage fractions from healthy human donors - depleted of bacteria and eukaryotic viruses - into high-fat-diet-fed mice, has led to improvements in liver histology and reshaping of the intestinal microbial community structure. These favorable outcomes suggest that phages may not only act through elimination of specific pathobionts, but also through more generalized modulation of microbiota-host interactions^[104]^.

In AH, a mechanistically analogous but clinically distinct phage therapy has been proposed, targeting cytolysin-producing *E. faecalis* strains that drive disease progression. Cytolysin, a two-component pore-forming exotoxin, disrupts hepatocyte membranes and activates immune responses. About one-third of severe AH patients carry cytolysin-positive *E. faecalis* strains, which are associated with worsened liver function and reduced survival. In humanized mouse models, oral delivery of cytolysin-specific lytic phages significantly reduced hepatic necrosis and inflammation, while non-specific phages showed no benefit, highlighting the need for strain-level targeting. Notably, phages were rapidly detected in systemic tissues - especially in ethanol-fed mice with increased gut permeability - and were cleared within 24 h without causing tissue damage^[29]^. Building upon these findings, a rationally designed phage cocktail comprising three lytic phages demonstrated lytic activity against approximately 75% of cytolysin-positive *E. faecalis* clinical isolates, suggesting broad applicability across patient populations. An ongoing clinical trial assessing cytolysin status and its prognostic implications in AH patients is expected to yield crucial insights that will inform the rational incorporation of *E. faecalis*-targeted phage therapy into microbiota-modifying treatment paradigms for alcohol-associated liver disease^[105]^.

### Cholangitis

*K. pneumoniae* strains enriched in PSC patient cohorts have been functionally implicated as major drivers of hepatic inflammation through Th17-mediated immune pathways. Building upon these insights, preclinical efforts have demonstrated that lytic phages specifically tailored to PSC-derived *K. pneumoniae* isolates retain robust killing efficiency across varying physicochemical conditions and maintain high host specificity. *In vivo* administration of these phage cocktails, delivered via both oral and systemic routes in mouse models, significantly decreased intestinal *K. pneumoniae* colonization (*P* < 0.05). This microbial reduction translated into marked improvements in liver histopathology, including reduced Th17 cell infiltration and improved inflammatory scores. Importantly, phage therapy preserved the overall structure of the gut microbiota and spared non-target commensals, highlighting its potential for targeted microbial modulation without collateral dysbiosis^[70]^. Moreover, adding to the therapeutic arsenal, a novel phage named Phi_Eg_SY1 has been isolated for its ability to lyse *E. gallinarum* strains derived from murine feces. This phage demonstrates strong strain-level specificity, tolerance to environmental stressors, and an absence of lysogeny-related or virulence genes, underscoring its biosafety credentials^[106]^. Although *in vivo* evaluation remains forthcoming, these findings support the rationale for incorporating *E. gallinarum*-directed lytic agents into broader phage cocktails aimed at mitigating pathobiont-driven inflammation in PSC.

In contrast to PSC, where multiple phage candidates have undergone experimental validation, research on phage therapy in PBC remains largely exploratory. Nonetheless, mechanistic studies have pointed to *E. gallinarum* as a key pathobiont capable of translocating from the gut lumen to the liver, where it promotes portal inflammation and contributes to the breakdown of immune tolerance. In gnotobiotic models, *E. gallinarum* not only recapitulated immunopathological features of autoimmune cholangitis but also acted synergistically with *K. pneumoniae* and *Proteus mirabilis* to exacerbate hepatic Th17 responses. These interactions suggest a complex microbial network contributing to disease progression, wherein *E. gallinarum* plays a central, amplifying role.

### Pancreatitis

Pancreatitis often involves gut-derived bacteria such as *Enterococcus* *spp*., *E. coli*, and *K. pneumoniae*, which translocate across a compromised intestinal barrier and contribute to infected pancreatic necrosis (IPN) and systemic inflammation. In severe cases, especially when MDR strains are involved, these infections substantially increase morbidity and mortality and remain difficult to manage using conventional antibiotics^[107-109]^.

A personalized phage cocktail, administered intravenously and directly into the infected cavity, successfully treated MDR *A. baumannii* in necrotizing pancreatitis after antibiotic therapy had failed, leading to full recovery^[98]^. This case exemplifies both the tissue-penetrating capacity of phages and their potential as adjuncts in severe pancreatic infections. Despite these advances, phage therapy in pancreatitis remains at an early exploratory stage. Future efforts should prioritize preclinical validation in pancreatic models, careful assessment of phage pharmacokinetics in necrotic tissues, and the design of precision-targeted cocktails informed by metagenomic profiling. Integration with conventional therapies, including antibiotics or microbiota-based interventions, may further enhance efficacy and safety.

Together, these studies underscore the therapeutic potential of phage-based strategies in modulating disease-relevant microbial and immune landscapes.

### Early human trials and safety evaluation

For most gastrointestinal diseases discussed here, except for CD and pancreatitis, clinical evidence on phage therapy remains scarce, and no standardized therapeutic protocols are currently available. Nevertheless, several early human studies have provided important insights into safety, tolerability, and translational barriers.

One of the earliest controlled trials tested oral administration of T4 coliphages in healthy adults at doses of 10^3^-10^5^ plaque-forming unit (PFU)/mL. Phages were recovered in stool in a dose-dependent manner without altering commensal *E. coli* populations, and no adverse events or systemic immune responses were observed, indicating that phages can safely transit the gastrointestinal tract^[110]^. Extending these observations to patients, a randomized trial in children with acute bacterial diarrhea evaluated oral phage cocktails. Although no therapeutic benefit was achieved - likely due to low pathogen abundance and incomplete phage coverage - the regimen was safe, with no phage-related adverse events or induction of anti-phage immunity^[97]^. These findings highlight both the favorable safety profile of oral phages and the limitations of efficacy in heterogeneous infectious contexts.

Subsequent placebo-controlled studies in healthy populations reinforced these results. A four-week intervention with a commercial *E. coli*-targeting phage product was well tolerated, with no gastrointestinal discomfort, systemic inflammation, or antibody responses. Phage intake selectively reduced fecal *E. coli* abundance without disturbing overall microbiota diversity, suggesting a role for phages as safe precision modulators of gut ecology rather than broad-spectrum antimicrobials^[111]^.

Beyond oral delivery, compassionate use cases have demonstrated the feasibility of phage therapy in critical care. In the previously described case of a MDR *A. baumannii* pancreatic infection, a personalized phage cocktail was administered both intravenously and via intracavitary delivery. Apart from a transient febrile episode attributed to sepsis rather than phage toxicity, no severe adverse events occurred, and the patient ultimately recovered. This case provided compelling clinical evidence linking phage therapy to the successful resolution of life-threatening pancreatitis-associated infections^[112]^.

Most recently, a phase I randomized trial (NCT04737876) tested an oral five-phage cocktail targeting *K. pneumoniae* in healthy adults. Twice-daily dosing for three days (2.8 × 10^10^ PFU per dose) was well tolerated, with no adverse effects, systemic absorption, or anti-phage antibody responses detected. Gut microbiota composition remained stable, while functional assays confirmed phage persistence and selective reduction of target strains. Although not designed to assess efficacy, this study represents an important regulatory milestone, showing that standardized phage formulations can be evaluated under clinical trial frameworks^[55]^.

Taken together, early human studies indicated that phage administration was generally safe across delivery routes, age groups, and disease contexts. The lack of efficacy in pediatric diarrhea trials highlighted specific translational barriers - including inadequate dosing, narrow host range, and variable pathogen burden - that needed to be addressed in trial design. In contrast, successful compassionate use cases and phase I studies demonstrated that phages could be applied to humans without major safety concerns. Together, these data provided a robust safety basis for advancing clinical evaluation of phage therapy in CD, pancreatitis, and other gastrointestinal disorders.

## CHALLENGES AND FUTURE DIRECTIONS

Despite the growing enthusiasm surrounding phage therapy as a precision tool to modulate gut microbial communities, translating this promising strategy into clinical practice for inflammatory gastrointestinal diseases continues to face substantial and multifaceted challenges. Bacteriophages exhibit unique therapeutic advantages, including exceptional specificity, self-replication within bacterial targets, and minimal off-target toxicity, but the intricate and dynamic interactions among host, microbiome, and phages within the inflamed gut necessitate a cautious, comprehensive, and multidisciplinary approach to clinical development.

One of the foremost barriers to successful clinical translation lies in the inherently narrow host range of bacteriophages. While this characteristic enables precise targeting and eradication of pathogenic bacterial strains, it simultaneously restricts therapeutic effectiveness in the complex, polymicrobial environments typically encountered in IBD^[113]^. Effective treatment strategies often require personalized phage cocktails meticulously tailored to the individual patient’s unique microbial dysbiosis. Such bespoke regimens increase manufacturing and regulatory complexity, as each lot requires defined release criteria for potency, purity, and stability^[19]^. Additionally, the marked microbial heterogeneity observed among IBD patients - further exacerbated by dynamic shifts during disease flare-ups or following antibiotic intervention - can significantly compromise sustained therapeutic outcomes and complicate the consistent identification of suitable microbial targets^[114]^. To overcome these limitations, high-throughput phage-host mapping has enabled rapid identification of phages matched to patient isolates, while clustered, regularly interspaced, short palindromic repeats (CRISPR)-based engineering can modify receptor-binding domains or restore activity against resistant strains. These strategies expand therapeutic coverage within dysbiotic communities and make individualized phage design more practical^[115,116]^.

Further compounding these difficulties is the rapid evolution of bacterial resistance. Bacteria can develop resistance to phages through various mechanisms, including mutations in phage receptor sites, deployment of restriction-modification systems, or activation of CRISPR-Cas immune defenses. Although the emergence of phage resistance may sometimes incur fitness costs to bacteria, it can also lead to unintended changes in bacterial virulence or facilitate horizontal gene transfer, potentially raising broader ecological safety concerns^[117]^. Consequently, clinical regimens often require iterative adaptation (re-selection or re-isolation), complicating bedside logistics and supply. To counter these dynamics, engineered phages are being developed to bypass bacterial defenses and suppress the emergence of resistance. Strategies include swapping or diversifying tail fiber proteins to broaden receptor recognition, encoding anti-CRISPR elements that neutralize bacterial adaptive immunity, and delivering CRISPR-Cas payloads designed to cleave resistance or virulence genes. In addition, directed evolution of receptor-binding proteins offers a complementary route to restore phage infectivity against escape variants, expanding therapeutic flexibility in rapidly evolving microbial communities^[118,119]^.

Beyond resistance mechanisms, phage-mediated horizontal gene transfer presents another layer of biosafety concern. In polymicrobial and inflamed environments, generalized or specialized transduction can facilitate the spread of antibiotic resistance genes and virulence factors between bacteria. Moreover, repeated rounds of phage amplification may promote genetic drift through mutation and recombination, potentially affecting host specificity or the stability of therapeutic traits^[120]^. To mitigate these risks, it is essential to perform thorough genomic screening of candidate phages, especially focusing on antimicrobial resistance determinants, virulence factors, and transduction potential. The use of strictly lytic, non-transducing phages, combined with post-production genome validation and stability assays, can help reduce the likelihood of unintended gene transfer and batch-to-batch variability^[121]^.

Although bacteriophages were long considered immunologically inert, recent studies have shown that they can elicit neutralizing antibody responses, especially after repeated administration or exposure via systemic or mucosal routes, thereby reducing their bioavailability and narrowing their therapeutic window^[122,123]^. Moreover, phage particles or bacterial lysis byproducts, such as endotoxins, may act as pathogen-associated molecular patterns (PAMPs). In mucosally compromised or inflamed environments, these factors can exacerbate inflammatory responses, potentially aggravating underlying pathology. Preclinical studies have shown that filamentous bacteriophages, in particular, can modulate immune signaling pathways, activating type I interferon responses or disrupting the balance between pro- and anti-inflammatory cytokines. Under certain disease conditions, such immune modulations may inadvertently intensify disease progression^[124]^. Nevertheless, several safer administration strategies are currently under investigation. For example, mucosa-targeted local delivery routes, such as oral or rectal administration, may help reduce systemic exposure. In addition, shortening the treatment duration or applying phage rotation strategies could delay the emergence of neutralizing antibodies and help sustain therapeutic efficacy^[125]^.

The practicalities associated with phage delivery further complicate clinical translation. Oral administration is widely regarded as optimal for intestinal disorders; however, the acidic gastric environment significantly reduces phage viability and therapeutic efficacy. Various encapsulation techniques, such as enteric-coated capsules, alginate-based beads, and hydrogels, have shown promise in protecting phages during gastric transit. Yet, interindividual variability in gastric pH, digestive enzyme activity, and intestinal transit time continues to complicate the pharmacokinetics and pharmacodynamics of orally delivered phages^[126,127]^. Moreover, effective delivery to inflamed intestinal regions, where mucosal integrity is often severely compromised and immune infiltration is highest, requires advanced targeting technologies, many of which remain experimental or under active development.

Perhaps most critically, the long-term ecological and immunological consequences of manipulating the human gut virome remain inadequately understood. The gut virome is increasingly recognized as a crucial component of microbial homeostasis, essential for host immune system development, metabolic regulation, and overall microbiota stability. Notably, recent studies have shown that transplantation of viromes from UC patients into germ-free mice exacerbated experimental colitis severity, suggesting that untargeted or misdirected phage interventions may inadvertently induce dysbiosis or immune dysregulation^[53]^. These findings underscore the need for precision not only in bacterial strain targeting but also in the genomic and functional selection of phages, taking into account their integration within broader microbial and immune networks.

Clinical translation is also hindered by the absence of standardized protocols for the preparation, characterization, and quality control of therapeutic phage products. Unlike conventional antibiotics, phages are self-replicating and evolving biological entities, challenging existing drug regulatory and classification frameworks. Regulatory agencies such as the US Food and Drug Administration (FDA) and European Medicines Agency (EMA) currently manage phage therapies largely under experimental or compassionate use provisions. This fragmented oversight complicates the development of harmonized safety, efficacy, and manufacturing standards, limiting large-scale clinical trials and deterring commercial investment - thereby substantially slowing therapeutic validation and market readiness^[128]^. Therefore, a coherent regulatory framework will be required to support clinical translation. Priorities include fast-track approval pathways, good manufacturing practice (GMP)-compliant phage banks, and regionally coordinated standards for production and quality control, all of which are necessary for large-scale and compliant implementation of phage therapy^[129]^.

Several biotechnology companies are actively advancing phage therapy from laboratory research to clinical application, reflecting growing regulatory support and commercial interest. Intralytix has received FDA clearance for an Investigational New Drug (IND) application and initiated a Phase I/IIa trial of EcoActive, an oral phage cocktail targeting adherent-invasive *E. coli* in CD^[130]^, representing one of the few ongoing programs directly focused on gastrointestinal disorders. In parallel, BiomX is developing phage cocktails for chronic inflammatory conditions; notably, an oral phage therapy targeting *Klebsiella* in IBD demonstrated safety in a Phase I study and showed potential for targeted microbiome modulation^[131]^. Collectively, these developments highlight the significant translational momentum of phage therapeutics and the increasing engagement of regulatory agencies. Moreover, industry consolidation and investment - illustrated by BiomX’s acquisition of Adaptive Phage Therapeutics in 2024 - further underscore that the field is steadily progressing toward commercial maturity^[132]^.

In conclusion, despite phage therapy’s significant conceptual promise for managing chronic gastrointestinal inflammation, its successful clinical adoption depends on overcoming a diverse array of biological, immunological, technological, and regulatory barriers. Future research must prioritize the elucidation of phage-microbiome-host interactions, refinement of adaptive phage engineering, optimization of delivery systems, and establishment of comprehensive safety evaluation frameworks. Bridging these domains will be essential to fully realize the therapeutic potential of bacteriophages in the management of inflammatory gastrointestinal diseases.

## CONCLUSION

The gut virome, particularly bacteriophages, is gaining increasing recognition as a key regulator of microbial balance and immune responses across a wide range of gastrointestinal and hepatopancreatic diseases, including IBD, MASH, AH, PSC, PBC, and pancreatitis. Common alterations, such as reduced viral diversity and increased phage targeting of specific pathobionts, suggest that phages actively contribute to disease onset and progression. On the therapeutic front, bacteriophages offer unique potential. Unlike broad-spectrum antibiotics, phages can precisely target pathogenic bacteria while sparing commensals. Preclinical and early clinical evidence, particularly in IBD and chronic liver diseases, has highlighted the therapeutic potential of phages. Building on these findings, applications are now expanding to conditions such as pancreatitis and cholangitis, with growing interest in microbiota-targeted strategies.

Nonetheless, the clinical development of phage therapy remains at an early stage. Several hurdles remain, including optimizing delivery, elucidating phage–host immune interactions, and managing resistance. Synthetic biology and microbiome science are paving new paths forward, including recombinant phages with enhanced specificity or controllable lytic activity. Additionally, innovative delivery systems, such as liposomes, hydrogels, and colon-targeted platforms, are being developed to improve phage stability and mucosal targeting. Integrating microbiome-informed diagnostics with high-throughput phage screening may accelerate personalized therapy and regulatory translation. Moving forward, rigorous mechanistic research and well-designed clinical trials will be pivotal to fully realize the therapeutic potential of this long-overlooked component of the gut virome.

## DECLARATIONS

### Copyright

© The Author(s) 2025.

## References

[1] Zheng D, Liwinski T, Elinav E (2020). Interaction between microbiota and immunity in health and disease. Cell Res..

[2] Wu J, Wang K, Wang X, Pang Y, Jiang C (2020). The role of the gut microbiome and its metabolites in metabolic diseases. Protein Cell..

[3] Thursby E, Juge N (2017). Introduction to the human gut microbiota. Biochem J..

[4] Michaudel C, Sokol H (2020). The gut microbiota at the service of immunometabolism. Cell Metab..

[5] Isenring J, Bircher L, Geirnaert A, Lacroix C (2023). *In vitro* human gut microbiota fermentation models: opportunities, challenges, and pitfalls. Microbiome Res Rep..

[6] Lozupone CA, Stombaugh JI, Gordon JI, Jansson JK, Knight R (2012). Diversity, stability and resilience of the human gut microbiota. Nature..

[7] Ni J, Wu GD, Albenberg L, Tomov VT Gut microbiota and IBD: causation or correlation? *Nat Rev Gastroenterol Hepatol.* 2017;14:573-84.

[8] Norman JM, Handley SA, Baldridge MT (2015). Disease-specific alterations in the enteric virome in inflammatory bowel disease. Cell..

[9] Tripathi A, Debelius J, Brenner DA (2018). The gut-liver axis and the intersection with the microbiome. Nat Rev Gastroenterol Hepatol..

[10] Vich Vila A, Collij V, Sanna S (2020). Impact of commonly used drugs on the composition and metabolic function of the gut microbiota. Nat Commun..

[11] Wong SH, Yu J (2019). Gut microbiota in colorectal cancer: mechanisms of action and clinical applications. Nat Rev Gastroenterol Hepatol..

[12] Young VB (2017). The role of the microbiome in human health and disease: an introduction for clinicians. BMJ..

[13] Vilà-quintana L, Fort E, Pardo L (2024). Metagenomic study reveals phage-bacterial interactome dynamics in gut and oral microbiota in pancreatic diseases. Int J Mol Sci..

[14] Chu H, Duan Y, Lang S (2020). The Candida albicans exotoxin candidalysin promotes alcohol-associated liver disease. J Hepatol..

[15] Lang S, Duan Y, Liu J (2019). Intestinal fungal dysbiosis and systemic immune response to fungi in patients with alcoholic hepatitis. Hepatology..

[16] Clooney AG, Sutton TD, Shkoporov AN (2019). Whole-virome analysis sheds light on viral dark matter in inflammatory bowel disease. Cell Host Microbe..

[17] Lang S, Demir M, Martin A (2020). Intestinal virome signature associated with severity of nonalcoholic fatty liver disease. Gastroenterology..

[18] Stockdale SR, Shkoporov AN, Khokhlova EV (2023). Interpersonal variability of the human gut virome confounds disease signal detection in IBD. Commun Biol..

[19] Tun HM, Peng Y, Massimino L (2024). Gut virome in inflammatory bowel disease and beyond. Gut..

[20] Zuo T, Lu XJ, Zhang Y (2019). Gut mucosal virome alterations in ulcerative colitis. Gut..

[21] Liang G, Bushman FD (2021). The human virome: assembly, composition and host interactions. Nat Rev Microbiol..

[22] Ackermann HW, Prangishvili D (2012). Prokaryote viruses studied by electron microscopy. Arch Virol..

[23] Twort FW (2009). Further Investigations on the nature of ultra-microscopic viruses and their cultivation. J Hyg (Lond)..

[24] Knowles B, Silveira CB, Bailey BA (2016). Lytic to temperate switching of viral communities. Nature..

[25] Davies EV, Winstanley C, Fothergill JL, James CE, Millard A (2016). The role of temperate bacteriophages in bacterial infection. FEMS Microbiol Lett..

[26] Olszak T, Latka A, Roszniowski B, Valvano MA, Drulis-kawa Z (2017). Phage life cycles behind bacterial biodiversity. Curr Med Chem..

[27] Dion MB, Oechslin F, Moineau S (2020). Phage diversity, genomics and phylogeny. Nat Rev Microbiol..

[28] Gan L, Feng Y, Du B (2023). Bacteriophage targeting microbiota alleviates non-alcoholic fatty liver disease induced by high alcohol-producing Klebsiella pneumoniae. Nat Commun..

[29] Duan Y, Llorente C, Lang S (2019). Bacteriophage targeting of gut bacterium attenuates alcoholic liver disease. Nature..

[30] Cao Z, Fan D, Sun Y (2024). The gut ileal mucosal virome is disturbed in patients with Crohn’s disease and exacerbates intestinal inflammation in mice. Nat Commun..

[31] Salmond GPC, Fineran PC (2015). A century of the phage: past, present and future. Nat Rev Microbiol..

[32] Aminov R (2017). History of antimicrobial drug discovery: major classes and health impact. Biochem Pharmacol..

[33] Becattini S, Taur Y, Pamer EG (2016). Antibiotic-Induced changes in the Intestinal Microbiota and Disease. Trends Mol Med..

[34] Goossens H, Ferech M, Vander Stichele R, Elseviers M (2005). Outpatient antibiotic use in Europe and association with resistance: a cross-national database study. Lancet..

[35] Spellberg B, Bartlett JG, Gilbert DN (2013). The future of antibiotics and resistance. N Engl J Med..

[36] Aranaga C, Pantoja LD, Martínez EA, Falco A (2022). Phage therapy in the era of multidrug resistance in bacteria: a systematic review. Int J Mol Sci..

[37] Hatfull GF, Dedrick RM, Schooley RT (2022). Phage therapy for antibiotic-resistant bacterial infections. Annu Rev Med..

[38] Uchechukwu CF, Shonekan A (2024). Current status of clinical trials for phage therapy. J Med Microbiol..

[39] Summers WC (2001). Bacteriophage therapy. Annu Rev Microbiol..

[40] Jiang L, Lang S, Duan Y (2020). Intestinal virome in patients with alcoholic hepatitis. Hepatology..

[41] Alkhalil SS (2023). The role of bacteriophages in shaping bacterial composition and diversity in the human gut. Front Microbiol..

[42] Rybicka I, Kaźmierczak Z, Vives M (2025). The human phageome: niche-specific distribution of bacteriophages and their clinical implications. Appl Environ Microbiol..

[43] Shuwen H, Kefeng D (2022). Intestinal phages interact with bacteria and are involved in human diseases. Gut Microbes..

[44] Abraham C, Cho JH (2009). Inflammatory bowel disease. N Engl J Med..

[45] Baumgart DC, Sandborn WJ (2012). Crohn’s disease. Lancet..

[46] Danese S, Fiocchi C (2011). Ulcerative colitis. N Engl J Med..

[47] Turner JR (2009). Intestinal mucosal barrier function in health and disease. Nat Rev Immunol..

[48] Clayburgh DR, Shen L, Turner JR (2004). A porous defense: the leaky epithelial barrier in intestinal disease. Lab Invest..

[49] Tian X, Li S, Wang C (2024). Gut virome-wide association analysis identifies cross-population viral signatures for inflammatory bowel disease. Microbiome..

[50] Majzoub ME, Paramsothy S, Haifer C (2024). The phageome of patients with ulcerative colitis treated with donor fecal microbiota reveals markers associated with disease remission. Nat Commun..

[51] Fernandes MA, Verstraete SG, Phan TG (2019). Enteric virome and bacterial microbiota in children with ulcerative colitis and Crohn disease. J Pediatr Gastroenterol Nutr..

[52] Wagner J, Maksimovic J, Farries G (2013). Bacteriophages in gut samples from pediatric Crohn’s disease patients: metagenomic analysis using 454 pyrosequencing. Inflamm Bowel Dis..

[53] Sinha A, Li Y, Mirzaei MK (2022). Transplantation of bacteriophages from ulcerative colitis patients shifts the gut bacteriome and exacerbates the severity of DSS colitis. Microbiome..

[54] Carasso S, Zaatry R, Hajjo H (2024). Inflammation and bacteriophages affect DNA inversion states and functionality of the gut microbiota. Cell Host Microbe..

[55] Federici S, Kredo-russo S, Valdés-mas R (2022). Targeted suppression of human IBD-associated gut microbiota commensals by phage consortia for treatment of intestinal inflammation. Cell..

[56] Gogokhia L, Buhrke K, Bell R (2019). Expansion of bacteriophages is linked to aggravated intestinal inflammation and colitis. Cell Host Microbe..

[57] Obermeier F, Dunger N, Strauch UG (2005). CpG motifs of bacterial DNA essentially contribute to the perpetuation of chronic intestinal inflammation. Gastroenterology..

[58] Loomba R, Sanyal AJ (2013). The global NAFLD epidemic. Nat Rev Gastroenterol Hepatol..

[59] Younossi Z, Anstee QM, Marietti M (2017). Global burden of NAFLD and NASH: trends, predictions, risk factors and prevention. Nat Rev Gastroenterol Hepatol..

[60] Hagström H, Shang Y, Hegmar H, Nasr P (2024). Natural history and progression of metabolic dysfunction-associated steatotic liver disease. Lancet Gastroenterol Hepatol..

[61] Hsu CL, Lang S, Demir M, Fouts DE, Stärkel P, Schnabl B (2023). Any alcohol use in NAFLD patients is associated with significant changes to the intestinal virome. Hepatology..

[62] Kwan SY, Sabotta CM, Cruz LR (2024). Gut phageome in Mexican Americans: a population at high risk for metabolic dysfunction-associated steatotic liver disease and diabetes. mSystems..

[63] Jew MH, Hsu CL (2023). Alcohol, the gut microbiome, and liver disease. J Gastroenterol Hepatol..

[64] Hsu CL, Zhang X, Jiang L (2022). Intestinal virome in patients with alcohol use disorder and after abstinence. Hepatol Commun..

[65] Llorente C, Jepsen P, Inamine T (2017). Gastric acid suppression promotes alcoholic liver disease by inducing overgrowth of intestinal Enterococcus. Nat Commun..

[66] Liwinski T, Heinemann M, Schramm C (2022). The intestinal and biliary microbiome in autoimmune liver disease - current evidence and concepts. Semin Immunopathol..

[67] Shah A, Macdonald GA, Morrison M, Holtmann G (2020). Targeting the gut microbiome as a treatment for primary sclerosing cholangitis: a conceptional framework. Am J Gastroenterol..

[68] Hov JR, Karlsen TH (2022). The microbiota and the gut-liver axis in primary sclerosing cholangitis. Nat Rev Gastroenterol Hepatol..

[69] Karlsen TH, Folseraas T, Thorburn D, Vesterhus M (2017). Primary sclerosing cholangitis - a comprehensive review. J Hepatol..

[70] Ichikawa M, Nakamoto N, Kredo-russo S (2023). Bacteriophage therapy against pathological Klebsiella pneumoniae ameliorates the course of primary sclerosing cholangitis. Nat Commun..

[71] Nakamoto N, Sasaki N, Aoki R (2019). Gut pathobionts underlie intestinal barrier dysfunction and liver T helper 17 cell immune response in primary sclerosing cholangitis. Nat Microbiol..

[72] Webb G, Siminovitch K, Hirschfield G (2015). The immunogenetics of primary biliary cirrhosis: A comprehensive review. J Autoimmun..

[73] Sarcognato S, Sacchi D, Grillo F (2021). Autoimmune biliary diseases: primary biliary cholangitis and primary sclerosing cholangitis. Pathologica..

[74] Xiang X, Yang X, Shen M (2021). Ursodeoxycholic acid at 18-22 mg/kg/d showed a promising capacity for treating refractory primary biliary cholangitis. Can J Gastroenterol Hepatol..

[75] Barba Bernal R, Ferrigno B, Morales EM (2023). Beth Israel Deaconess Medical Center, Boston, MA, USA. Management of primary biliary cholangitis: current treatment and future perspectives. Turk J Gastroenterol..

[76] Tang R, Wei Y, Li Y (2018). Gut microbial profile is altered in primary biliary cholangitis and partially restored after UDCA therapy. Gut..

[77] Trivedi PJ, Hirschfield GM, Adams DH, Vierling JM (2024). Immunopathogenesis of primary biliary cholangitis, primary sclerosing cholangitis and autoimmune hepatitis: themes and concepts. Gastroenterology..

[78] Guo Z, He K, Pang K (2024). Exploring advanced therapies for primary biliary cholangitis: insights from the gut microbiota-bile acid-immunity network. Int J Mol Sci..

[79] Lankisch PG, Apte M, Banks PA (2015). Acute pancreatitis. Lancet..

[80] Banks PA, Bollen TL, Dervenis C (2013). Classification of acute pancreatitis - 2012: revision of the Atlanta classification and definitions by international consensus. Gut..

[81] Beyer G, Habtezion A, Werner J, Lerch MM, Mayerle J (2020). Chronic pancreatitis. Lancet..

[82] Campion EW, Forsmark CE, Swaroop Vege S, Wilcox CM (2016). Acute pancreatitis. N Engl J Med..

[83] Tan C, Ling Z, Huang Y (2015). Dysbiosis of intestinal microbiota associated with inflammation involved in the progression of acute pancreatitis. Pancreas..

[84] Frost F, Weiss FU, Sendler M (2020). The gut microbiome in patients with chronic pancreatitis is characterized by significant dysbiosis and overgrowth by opportunistic pathogens. Clin Transl Gastroenterol..

[85] Zhang C, Li G, Lu T (2023). The interaction of microbiome and pancreas in acute pancreatitis. Biomolecules..

[86] Zhu Y, Mei Q, Fu Y, Zeng Y (2021). Alteration of gut microbiota in acute pancreatitis and associated therapeutic strategies. Biomed Pharmacother..

[87] Shareefdeen H, Hill C (2022). The gut virome in health and disease: new insights and associations. Curr Opin Gastroenterol..

[88] Schepis T, De Lucia SS, Nista EC (2021). Microbiota in pancreatic diseases: a review of the literature. J Clin Med..

[89] Patel BK, Patel KH, Bhatia M, Iyer SG, Madhavan K, Moochhala SM (2021). Gut microbiome in acute pancreatitis: a review based on current literature. World J Gastroenterol..

[90] Li J, Pan X, Yang J (2020). Enteral virus depletion modulates experimental acute pancreatitis via toll-like receptor 9 signaling. Biochem Pharmacol..

[91] Sinha A, Maurice CF (2019). Bacteriophages: uncharacterized and dynamic regulators of the immune system. Mediators Inflamm..

[92] (2001). Sulakvelidze A, Alavidze Z, Morris JG Jr. Bacteriophage therapy. Antimicrob Agents Chemother..

[93] Asheshov IN, Khan S, Lahiri MN

[94] Titécat M, Rousseaux C, Dubuquoy C (2022). Safety and efficacy of an AIEC-targeted bacteriophage cocktail in a mice colitis model. J Crohns Colitis..

[95] (2015). Sangster W, Hegarty JP, Stewart DB Sr. Phage tail-like particles kill Clostridium Difficile and represent an alternative to conventional antibiotics. Surgery..

[96] Corbellino M, Kieffer N, Kutateladze M (2020). Eradication of a multidrug-resistant, carbapenemase-producing klebsiella pneumoniae isolate following oral and intra-rectal therapy with a custom made, lytic bacteriophage preparation. Clin Infect Dis..

[97] Sarker SA, Sultana S, Reuteler G (2016). Oral phage therapy of acute bacterial diarrhea with two coliphage preparations: a randomized trial in children from Bangladesh. EBioMedicine..

[98] Schooley RT, Biswas B, Gill JJ (2017). Development and use of personalized bacteriophage-based therapeutic cocktails to treat a patient with a disseminated resistant Acinetobacter baumannii infection. Antimicrob Agents Chemother..

[99] Mah C, Jayawardana T, Leong G (2023). Assessing the relationship between the gut microbiota and inflammatory bowel disease therapeutics: a systematic review. Pathogens..

[100] Taylor VL, Fitzpatrick AD, Islam Z, Maxwell KL

[101] Guerrero-Bustamante CA, Hatfull GF, Ehrt S (2024). Bacteriophage tRNA-dependent lysogeny: requirement of phage-encoded tRNA genes for establishment of lysogeny. mBio..

[102] Manrique P, Montero I, Fernandez-gosende M, Martinez N, Cantabrana CH, Rios-covian D (2024). Past, present, and future of microbiome-based therapies. Microbiome Res Rep..

[103] Wang Q, Euler CW, Delaune A, Fischetti VA (2015). Using a novel lysin to help control Clostridium Difficile infections. Antimicrob Agents Chemother..

[104] Mao X, Larsen SB, Zachariassen LSF (2024). Transfer of modified gut viromes improves symptoms associated with metabolic syndrome in obese male mice. Nat Commun..

[105] Mendes BG, Duan Y, Schnabl B (2022). Immune response of an oral Enterococcus faecalis phage cocktail in a mouse model of ethanol-induced liver disease. Viruses..

[106] Chen Q, Dong Z, Ding T (2023). Isolation and characterization of a novel Enterococcus phage Phi_Eg_SY1. Virus Res..

[107] Badia JM, Amador S, González-sánchez C (2024). Appropriate use of antibiotics in acute pancreatitis: a scoping review. Antibiotics (Basel)..

[108] Lu J, Ding Y, Qu Y (2022). Risk factors and outcomes of multidrug-resistant bacteria infection in infected pancreatic necrosis patients. Infect Drug Resist..

[109] Hao H, Liu Y, Cao J (2021). Genomic new insights into emergence and clinical therapy of multidrug-resistant klebsiella pneumoniae in infected pancreatic necrosis. Front Microbiol..

[110] Bruttin A, Brüssow H (2005). Human volunteers receiving phage T4 orally: a safety test of phage therapy. Antimicrob Agents Chemother..

[111] Grubb DS, Wrigley SD, Freedman KE (2020). PHAGE-2 study: supplemental bacteriophages extend bifidobacterium animalis subsp. lactis BL04 benefits on gut health and microbiota in healthy adults. Nutrients..

[112] Liu M, Hernandez-morales A, Clark J (2022). Comparative genomics of Acinetobacter baumannii and therapeutic bacteriophages from a patient undergoing phage therapy. Nat Commun..

[113] Jansen D, Matthijnssens J (2023). The emerging role of the gut virome in health and inflammatory bowel disease: challenges, covariates and a viral imbalance. Viruses..

[114] Glassner KL, Abraham BP, Quigley EM (2020). The microbiome and inflammatory bowel disease. J Allergy Clin Immunol..

[115] Koncz M, Stirling T, Hadj Mehdi H (2024). Genomic surveillance as a scalable framework for precision phage therapy against antibiotic-resistant pathogens. Cell..

[116] Yehl K, Lemire S, Yang AC (2019). Engineering phage host-range and suppressing bacterial resistance through phage tail fiber mutagenesis. Cell..

[117] Oechslin F (2018). Resistance development to bacteriophages occurring during bacteriophage therapy. Viruses..

[118] Bikard D, Euler CW, Jiang W (2014). Exploiting CRISPR-Cas nucleases to produce sequence-specific antimicrobials. Nat Biotechnol..

[119] Gencay YE, Jasinskytė D, Robert C (2023). Engineered phage with antibacterial CRISPR-Cas selectively reduce E. coli burden in mice. Nat Biotechnol..

[120] Chen J, Quiles-puchalt N, Chiang YN (2018). Genome hypermobility by lateral transduction. Science..

[121] Penadés JR, Chen J, Quiles-puchalt N, Carpena N, Novick RP (2015). Bacteriophage-mediated spread of bacterial virulence genes. Curr Opin Microbiol..

[122] Berkson JD, Wate CE, Allen GB (2024). Phage-specific immunity impairs efficacy of bacteriophage targeting Vancomycin resistant Enterococcus in a murine model. Nat Commun..

[123] Łusiak-szelachowska M, Międzybrodzki R, Rogóż P, Weber-dąbrowska B, Żaczek M, Górski A (2022). Do anti-phage antibodies persist after phage therapy? A preliminary report. Antibiotics (Basel)..

[124] Liu D, Van Belleghem JD, De Vries CR (2021). The safety and toxicity of phage therapy: a review of animal and clinical studies. Viruses..

[125] Champagne-jorgensen K, Luong T, Darby T, Roach DR (2023). Immunogenicity of bacteriophages. Trends Microbiol..

[126] Colom J, Cano-sarabia M, Otero J (2017). Microencapsulation with alginate/CaCO_3_: a strategy for improved phage therapy. Sci Rep..

[127] Moghtader F, Solakoglu S, Piskin E (2024). Alginate- and chitosan-modified gelatin hydrogel microbeads for delivery of E. coli phages. Gels..

[128] Yang Q, Le S, Zhu T, Wu N (2023). Regulations of phage therapy across the world. Front Microbiol..

[129] Strathdee SA, Hatfull GF, Mutalik VK, Schooley RT (2023). Phage therapy: From biological mechanisms to future directions. Cell..

[130] https://www.prnewswire.com/news-releases/intralytix-receives-fda-clearance-to-initiate-phase-i--iia-clinical-trials-300599772.html.

[131] https://ir.biomx.com/news-events/press-releases/detail/42/biomx-announces-positive-results-of-a-phase-1a.

[132] https://ir.biomx.com/news-events/press-releases/detail/103/biomx-announces-closing-of-the-acquisition-of-adaptive.

